# Increased Migration of Human Mesenchymal Stromal Cells by Autocrine Motility Factor (AMF) Resulted in Enhanced Recruitment towards Hepatocellular Carcinoma

**DOI:** 10.1371/journal.pone.0095171

**Published:** 2014-04-15

**Authors:** Juan Bayo, Esteban Fiore, Jorge B. Aquino, Mariana Malvicini, Manglio Rizzo, Estanislao Peixoto, Oscar Andriani, Laura Alaniz, Flavia Piccioni, Marcela Bolontrade, Osvaldo Podhajcer, Mariana G. Garcia, Guillermo Mazzolini

**Affiliations:** 1 Gene Therapy Laboratory, Facultad de Ciencias Biomédicas, Universidad Austral, Derqui-Pilar, Buenos Aires, Argentina; 2 CONICET (Consejo Nacional de Investigaciones Científicas y Técnicas), Buenos Aires, Argentina; 3 Liver Unit, Hospital Universitario Austral, Universidad Austral, Derqui-Pilar, Argentina; 4 Molecular and Cellular Therapy Laboratory, Fundación Instituto Leloir, Buenos Aires, Argentina; Aix-Marseille University, France

## Abstract

**Background and Aims:**

Several reports described the migration of human mesenchymal stromal cells (MSCs) towards tumor-released factors. Autocrine motility factor (AMF) is produced by several tumors including hepatocellular carcinoma (HCC). The aim of this study was to analyze AMF involvement on MSC migration towards human HCC.

**Methods:**

Production of AMF by HCC tumors was evaluated by western analysis. The effects of AMF on MSCs from different sources (bone marrow, adipose tissue and perivascular cells from umbilical cord) were analyzed using *in vitro* migration assay; metalloproteinase 2 (MMP2) activity and expression of critical genes were studied by zymography and qRT-PCR, respectively. To assess AMF involvement on the *in vivo* MSC migration, noninvasive fluorescence imaging was performed. To test the effect of AMF-primed MSCs on tumor development, *in vitro* proliferation and spheroids growth and *in vivo* tumor volume were evaluated.

**Results:**

AMF produced by HCC was found to induce migration of different MSCs *in vitro* and to enhance their MMP2 activity. Stimulation of MSCs with recombinant AMF (rAMF) also induced the *in vitro* adhesion to endothelial cells in coincidence with changes in the expression levels of MMP3, AMF receptor, caveolin-1, and -2 and GDI-2. Importantly, stimulation of MSCs with rAMF increased the *in vivo* migration of MSCs towards experimental HCC tumors. AMF-priming of MSCs did not induce a pro-tumorigenic effect on HCC cells neither *in vivo* nor *in vitro*.

**Conclusion:**

AMF plays a role in MSC recruitment towards HCC. However, its ability to increase MSC migration to HCC for therapeutic purposes merits further evaluation.

## Introduction

Hepatocellular carcinoma (HCC) is the sixth most common cancer worldwide and the third cause of cancer-related death [1]. Curative therapies such as resection or liver transplantation improve patient survival [2]; however, these strategies can only be applied to a scarce minority of patients. Therefore, there is an urgent need of novel therapeutics for patients with advanced HCC.

Mesenchymal stromal cells (MSCs), also known as mesenchymal stem cells, constitute a heterogeneous cell population, characterized by their adherence to plastic, fibroblast-like morphology, expression of specific markers (CD105+, CD90+, CD73+), lack of hematopoietic markers (CD45, CD34, CD14 or CD11b, CD79α or CD19) and HLA class II, and capability to differentiate *in vitro* into osteoblasts, adipocytes and chondroblasts [3]. MSCs are most often derived from bone marrow (BM), but can also be isolated from adipose tissue (AT) or from umbilical cord; in the latter case, MSCs have been isolated from Wharton's jelly (WJ-MSCs), perivascular areas (HUCPVCs) or umbilical cord blood (CB-MSCs) [4]. MSCs show tropism for inflamed, injured or tumorigenic sites and this property together with their ability to be cultured and expanded *in vitro*, their self-renewal and their low immunogenicity make them good candidates for cell-based therapy [5]. Several reports described the use of MSCs as vehicles for therapeutic genes in cancer, exploiting the advantage of MSC homing into the tumor microenvironment [6]. However, mechanisms involved in MSC tumor recruitment are not fully understood.

Autocrine motility factor (AMF) is a 55-kDa cytokine secreted by tumors that autocrinally regulates cell motility [7]. AMF exhibits sequence identity with glucose-6-phosphate isomerase (GPI), a glycolytic enzyme involved in carbohydrate metabolism [8]. In tumor cells, the stimulation of cell motility was shown to be induced by the binding of AMF to its cognate receptor (AMFR), a 78-kDa seven transmembrane glycoprotein with leucine zipper and RING-H2 motifs [9]. AMFR is stably localized in caveolae, and caveolin-1 (CAV-1) has the ability to regulate the endocytic pathway through the stabilization of caveolae expression [10].

It was previously reported that AMF is secreted by different cancer types such as lung [11], gastrointestinal, kidney and breast [12] as well as HCC [13]. AMF-dependent stimulation of HCC has been associated with the upregulation of metalloproteinase 3 (MMP3) [14] and activation of the small G-protein RhoC [15]. Analysis of the signaling pathway triggered by AMF demonstrated phosphorylation of ERK 1/2 and JNK 1/2/3, leading to activation of c-Fos and c-Jun [16].

In this report, we describe for the first time the role of AMF in promoting MSC migration. Our results demonstrate that AMF produced by HCC is involved in migration of MSCs. Moreover, AMF was also able to induce MSC adhesion to endothelial cells and activation of MMPs *in vitro* as well as to increase MSC recruitment into HCC tumors *in vivo*.

## Materials and Methods

### Ethics statement

Animals were maintained at our Animal Resource Facilities (School of Biomedical Sciences, Austral University) in accordance with the experimental ethical committee and the NIH guidelines on the ethical use of animals. The “Animal Care Committee” from School of Biomedical Sciences, Austral University, approved the experimental protocol.

BM-MSCs, HUCPVCs and AT-MSCs were obtained from healthy donors after written informed consent and protocol were approved by the “Institutional Evaluation Committee” (CIE) from School of Biomedical Sciences, Austral University (Protocol No. 12-019).

### Cell lines

Human HCC cell line HuH7 were kindly provided by Prof. Jesus Prieto (CIMA, University of Navarra, Pamplona, Spain) [17]. LX-2 cell line (human hepatic stellate cells generated by spontaneous immortalization in low serum conditions) was kindly provided by Dr. Scott Friedman (Division of Liver Diseases, Mount Sinai School of Medicine, New York, NY, USA) [18]. Human microvascular endothelial cells (HMEC-1) were from CDC (Centers for Disease Control, Atlanta, GA, USA). Cell lines were cultured in complete DMEM (2 µM glutamine, 100 U/ml penicillin, 100 mg/ml streptomycin) and 10% heat-inactivated fetal bovine serum (FBS). Primary culture of HCC cells (HC-PT-5) was previously generated in our laboratory [19]. The collection of the sample and the project was approved by the Institutional Evaluation Committee (CIE) from School of Biomedical Sciences, Austral University (Protocol No. 11-007) and written informed consent was obtained from the patient. HC-PT-5 was cultured up to 8 passages in 70% DMEM/30% F12 (Invitrogen/Life Technologies) culture medium supplemented with 2 µM glutamine, 100 U/ml penicillin, 100 mg/ml streptomycin and 10% FBS.

### Isolation of BM-MSCs, AT-MSCs and HUCPVCs

BM-MSCs were obtained from healthy donors (Hospital Naval Pedro Mallo, Buenos Aires, Argentina) as described previously [19]. For AT-MSC generation, cells were isolated from discarded fat from liposuctions as previously described [20]. Briefly, lipoaspirated material was washed extensively with sterile phosphate-buffered saline and then treated with 0.075% type collagenase (Sigma-Aldrich) in PBS for 30 min at 37°C with agitation. Cells were centrifuged and pellet was plated in complete DMEM low glucose/20% FBS (Internegocios S.A., Argentina) and used for different experiments between passages 4 to 6.

HUCPVCs were isolated from umbilical cord obtained from healthy donors at the Hospital Universitario Austral (Pilar, Buenos Aires, Argentina) using a protocol adapted from Davies *et al*. [21]. In brief, umbilical cords were dissected and vessels with their surrounding Warthon's Jelly were pulled out. The perivascular Wharton's Jelly was removed from the vessels and mechanically disrupted. Minced fragments were plated in complete DMEM low glucose/20% FBS (Internegocios S.A., Argentina). After 7 days incubation, non-adherent cells and minced fragments were removed and adherents HUCPVCs were cultured and used for different experiments at passages 4 to 6.

MSCs were characterized according to the International Society for Cellular Therapy (ISCT) guidelines [3] (not shown).

### Conditioned Medium

To obtain tumor conditioned medium (TCM), HuH7 cells (2×10^6^) or HC-PT-5 cells (5×10^6^) were inoculated subcutaneously (SC) into the right flank of nude mice. When tumors reached 200 mm^3^ in size approximately, tumors were dissected and minced into pieces smaller than 1 mm^3^ and transferred to a 24-well tissue culture plate (6 fragments/well) with 500 µl of complete DMEM without FBS. Cell conditioned medium (CCM) was obtained from HCC cell lines cultured as previously described to 90% confluence and then washed with PBS and cultured with complete DMEM without FBS. In both cases, 18 hours later, conditioned media were harvested and stored at –80°C until use.

### Western Blot

BM-MSCs or AMF stimulated BM-MSCs (incubated overnight with 1 µg/ml of rAMF in DMEM) were lysed with 150 mM NaCl, 20 mM Tris–HCl, pH 7.4, 0.1% SDS, 1.0% Nonidet P-40, 0.5% Na-deoxycholate, 0.2 mM phenylmethylsulfonyl fluoride and protease inhibitor cocktail. Lysates were centrifuged at 12,000× g for 20 minutes and the supernatants were used as total cell lysates. CCM and TCM were 100-fold concentrated using Vivaspin 6 centrifugal concentrator (Sartorius-Stedim Biotech). Protein concentration was determined by Bradford protein assay (Bio-Rad). Protein was separated using SDS-PAGE and transferred onto nitrocellulose membrane (Hybond-ECL, Amersham Biosciences). Blots were blocked and incubated with anti-AMF polyclonal antibody (1∶700, sc-33777, Santa Cruz Biotechnology) and anti-AMFR (1∶500, AP2162a, ABGENT) at 4°C overnight. Finally, blots were incubated with the corresponding HRP-conjugated at room temperature for 1 hour. Reactions were visualized by chemiluminescence. Staining with colloidal Coomassie was performed as loading control for conditioned medium as previously reported [22]. Density of each band was quantified with Scion Image software (Scion Corporation, Frederick, MD).

### *In vitro* migration and invasion assays

*In vitro* migration was performed using a 48-Transwell microchemotaxis Boyden Chamber unit (Neuroprobe, Inc.) as previously described [19]. MSCs (1.2×10^3^ cells/well) were placed in the upper chamber and DMEM, TCM or rAMF were applied to the lower chamber of the transwell unit. Chemokinesis controls were performed placing rAMF in the upper and lower chamber. For blocking experiments, TCM were preincubated for 60 min with anti-AMF antibody or isotype control IgG. For AMF pretreatment, BM-MSCs were incubated overnight with 1 µg/ml of rAMF in DMEM without FBS, or DMEM without FBS as control. For the invasion assay the polycarbonate filters were previously incubated with 10 µg/ml type IV collagen (Sigma-Aldrich) for 18 h at 4°C and for MMP inhibition, BM-MSCs were preincubated with 1,10 phenantroline (0.5 or 1 mM) (Sigma-Aldrich). MSCs viability was not affected by 1,10 phenantroline (not shown). All systems were left for 4 hours at 37°C in a 5% CO_2_ humidified atmosphere. Cells attached to the lower side of the membrane were fixed in 2% formaldehyde, stained with 4′,6-diamidino-2-phenylindole dihydrochloride (DAPI, Sigma-Aldrich) and counted using fluorescent-field microscopy at 100X. Captured images from three representative visual fields were analyzed using CellProfiler software (www.cellprofiler.com), and the mean number of cells/field ±SEM was calculated. For wound-healing assay, Fast-DiO-stained MSCs were seeded at 2.5×10^4^ cell/cm^2^ in DMEM with 10% FBS for 24 hours. Then, cells were overnight preincubated with 1 µg/ml rAMF or DMEM without FBS. The monolayers were then scratched by a 200 µl-tip, washed with PBS and incubated for 24 hours more in DMEM without FBS. Cells within the scratched area were counted under a fluorescent-field microscopy at 40X and number of cells/field were represented. Additionally, adherent cells were counted at the end of the experiment confirming the same number of cells in all the conditions tested.

### Gelatin Zymography Assay

To evaluate whether AMF may be able to induce gelatinolytic activity in MSCs, 5×10^4^ cells were seeded in 24-well plates for 18 hours. Cells were treated with 1 µg/ml of rAMF, TCM or serum-free DMEM as untreated control for 2 hours; then, MSCs were washed with PBS and cultured in DMEM for 6 hours before supernatants were collected. For blocking experiments, TCM were preincubated for 60 minutes with anti-AMF antibody or isotype control IgG. MMP2 activity was determined by zymography as previously described [19]. Relative MMP2 activity scores were obtained by normalizing values to untreated samples (DMEM).

### Cell Adhesion Assays

For analyses of BM-MSC adhesion to endothelial cells, 2×10^5^ HMEC were seeded in 96-well microplates and cultured for 1 day prior the assay. Coated wells were incubated for 5 minutes with 0.1 ml of 5×10^4^ cells/ml of Fast-DiO prelabelled MSCs which were or not pretreated with 1 µg/ml rAMF. Cell suspension was discarded and attached cells were fixed with 2% paraformaldehyde. Cells were counted using fluorescent-field microscopy at 200X: pictures taken from ten representative visual fields were analyzed using CellProfiler software (www.cellprofiler.com) and values were normalized to untreated control.

### Reverse Transcription-polymerase Chain Reaction (RT-PCR)

Total RNA of BM-MSCs pretreated or not overnight with 1 µg/ml rAMF was extracted using Trizol Reagent (Sigma-Aldrich Co., St. Louis, MO). For quantification of MMP3 mRNA level, MSCs were FBS starved 24 hours before rAMF pretreatment. Total RNA (4 µg) was reverse transcribed with 200 U of SuperScript II Reverse Transcriptase (Invitrogen, Carlsbad, CA) using 500 ng of Oligo (dT) primers. cDNAs were subjected to real-time polymerase chain reaction (qPCR) (Stratagene Mx3005p, Stratagene, La Jolla, CA, USA). For qRT-PCR, the mRNA levels of metalloproteinase 3 (MMP3), AMF receptor (AMFR), GDP dissociation inhibitor 2 (GDI-2), caveolin-1 (CAV-1) caveolin-2 (CAV-2) were quantified by SYBR® Green (Invitrogen), using the following primers:

MMP3 forward 5′-ACGCCAGCCAACTGTGATCCT-3′ and reverse 5′-ATATGCGGCATCCACGCCTGAA-3′; AMFR forward 5′-ACAAGATGTGGGCCTTGCAAGA-3 and reverse 5′-AAAACGCAGTGCTCCCAGGATA-3′; GDI-2 forward 5′-GACCAGCTTTGGAGCTCTTG-3′ and reverse 5′-TGCGGGAAATAAAGATCTGG-3′; CAV-1 forward 5′-AATCCAAGCATCCCTTTGCCCA-3′ and reverse 5′- ACCAGGCAGCTTTCTGTACGA-3′; CAV-2 forward 5′-GAGAGACAGGGGAGTTGTCAACTT-3′ and reverse 5′- GCCCGGCCCAGAAATAATGAGAT-3′. PCR amplifications were carried out using a cycle of 95°C for 10 minutes and 45 cycles under the following parameters: 95°C for 30 seconds, 58°C for 30 seconds, 72°C for 1 minute. At the end of PCR reaction, the temperature was increased from 60°C to 95°C at a rate of 2°C/min, and the fluorescence was measured every 15 seconds to construct the melting curve. Values were normalized to levels of glyceraldehyde-3-phosphate dehydrogenase (GAPDH; used as housekeeping) transcript (forward 5′-CATCTCTGCCCCCTCTGCTG-3′ and reverse 5′-GCCTGCTTCACCACCTTCTTG-3′). Data were processed by the ΔΔCt method. The relative amount of the PCR product amplified from untreated cells was set as 1. A non-template control (NTC) was run in every assay, and all determinations were performed as triplicates in three separated experiments.

### Proliferation Assays

Cell proliferation was evaluated by [3H]- thymidine incorporation assay. Briefly, HCC cells were seeded in 96-well culture tissue plates at 3×10^4^ cells/cm^2^ density for 1 day prior to the assay. Then cells were cultured with CM obtained from BM-MSCs pretreated with 1 µg/ml of rAMF for 48 hours followed by a pulse of thymidine 18 hours before the end of the experiment. DMEM and CM from untreated BM-MSCs were used as control. Finally, thymidine incorporation was measured in a scintillation counter. Each sample was assayed in sextuplicates and normalized to DMEM as control.

### Three-Dimensional Spheroids

Ninety-six-well tissue culture plates were coated with 2% agarose in PBS. A total of 3×10^3^ HuH7 cells, 1×10^3^ LX-2, 1×10^3^ HMEC-1 with or without 1×10^3^ BM-MSCs per spheroid were mixed in complete DMEM to obtain a single multicellular spheroid per well. Seventy-five microliters of supernatant were carefully removed from each well every 2 days and replaced with fresh medium. Viability above 75% was confirmed by Trypan blue exclusion test in all experiments. Spheroid size was evaluated using inverted microscopy at 40X: images were captured and values determined using ImageJ software (National Institute of Health, NIH). Spheroid volume was calculated using the formula π/6× larger diameter × (smaller diameter)^2^, and expressed as arbitrary units.

### Mice and *in vivo* experiments

Six- to eight-week-old male nude BALB/c mice were purchased from CNEA (Comisión Nacional de Energía Atómica, Ezeiza, Buenos Aires, Argentina). SC HuH7 tumors (2×10^6^ cells) were established and 10 days later BM-MSCs or BM-MSCs pretreated with rAMF were intravenously (IV) injected. Tumor growth was assessed by calliper measurement, and tumor volume (mm^3^) was calculated by the formula π/6× larger diameter × (smaller diameter)^2^. For *in vivo* migration studies, BM-MSCs or BM-MSCs pretreated with rAMF (5×10^5^) were prelabeled with CMDiI for histological analysis and DiR (Molecular Probes, Invitrogen) for fluorescence imaging (FI). FI was performed using the Xenogen In Vivo Imaging System (IVIS; Caliper Life Sciences, Hopkinton, MA, USA) 1 hour after MSC injection and every day until experimental end point. Images represent the radiant efficiency and were analyzed with IVIS Living Image (Caliper Life Sciences) software. Regions of interest (ROI) were automatically drawn around the isolated organs to assess the fluorescence signal emitted. Results were expressed as average radiant efficiency in units of photons/second within the region of interest [p/s/cm^2^/sr]/[µW/cm^2^] or as total radiant efficiency in units of photons/second within the region of interest [p/s]/[µW/cm^2^].

### Detection of BM-MSCs by Fluorescence

To detect CMDiI+ cells within tumors, frozen sections were mounted in mounting media with DAPI (Vector Laboratories, Inc.) and observed under a fluorescence microscope at 200X.

### Statistical Analyses

Unpaired 2-sided Student's t test, one-way analysis of variance following by post tests or Kruskal-Wallis and Dunn's post-tests (GraphPad Prism Software) were used for statistical analyses. Differences with p values lower than 0.05 were considered as statistically significant.

## Results

### AMF produced by HCC induce migration of MSCs from different sources

**Figure 1:**
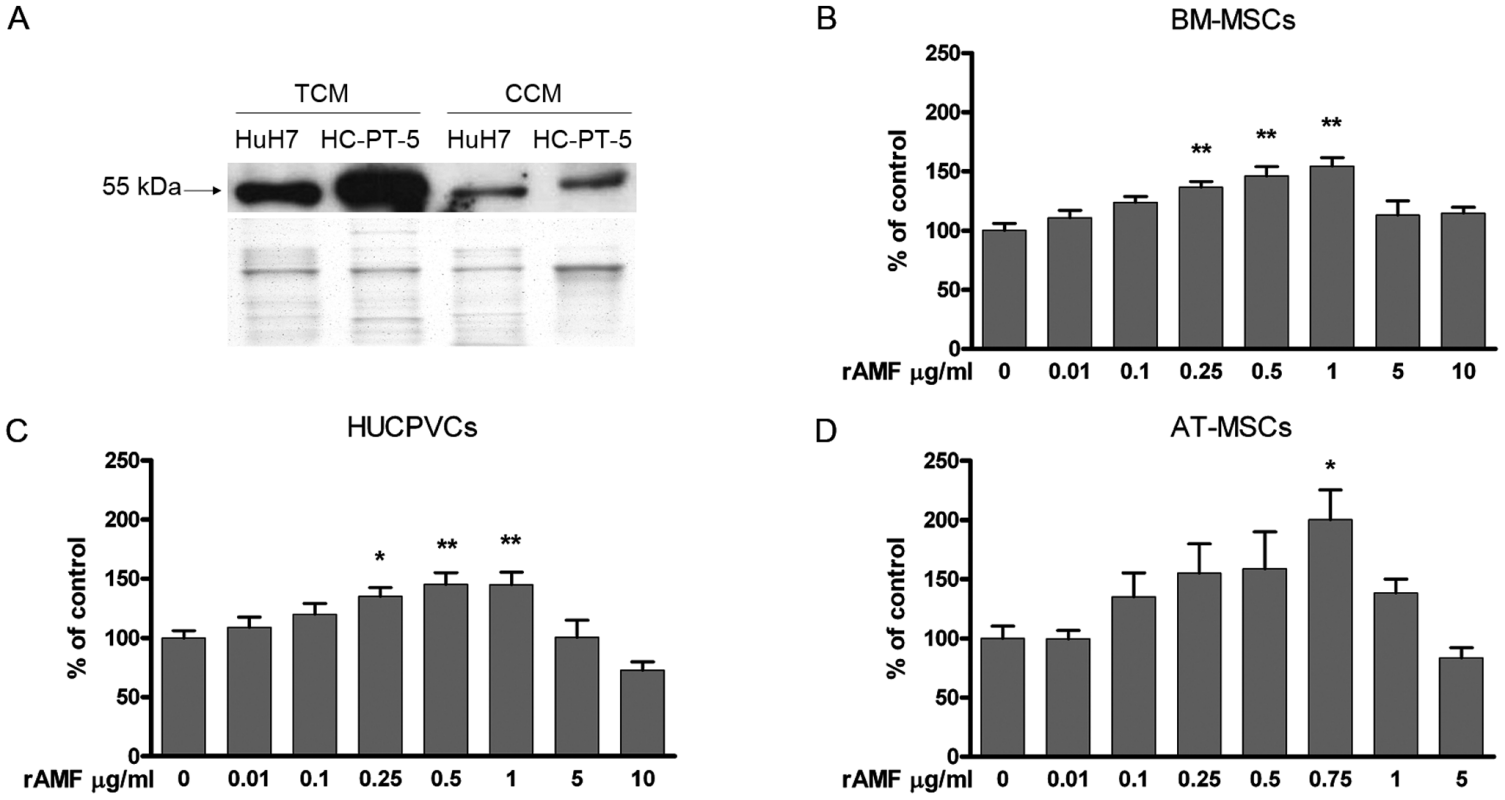
AMF potently stimulates in vitro chemotaxis of MSCs from different sources. A) Detection of AMF (55 kDa) by western blot in CCM derived from HCC cells and TCM from *ex vivo* HCC SC tumors (upper panel). Colloidal Coomassie staining was performed as loading control (lower panel). MSC migration was analyzed with a Boyden chamber assay using rAMF as chemoattractant for BM-MSCs (B), HUCPVCs (C) or AT-MSCs (D). Results were expressed as percentage of control (DMEM) ±SEM. *p<0.05 and **p<0.01 vs DMEM (ANOVA and Dunnett's test).

A 55 KDa soluble AMF form was detected in the conditioned medium of cell culture monolayers (CCM) and SC tumors (TCM) derived from HuH7 cell line and from HC-PT-5 HCC primary cultures (Figure 1A). AMF levels in TCM were higher when compared to CCM of similar origin. We then asked whether AMF may be able to act as MSC chemoattractant *in vitro*. With this aim, migration capacity of human MSCs derived from bone marrow (BM-MSCs), perivascular umbilical cord (HUCPVCs) and adipose tissue (AT-MSCs) towards AMF was analyzed using a modified Boyden chamber assay. MSCs isolated from the three sources studied migrated in a dose-dependent manner towards rAMF (Figure 1B–D). Chemokinesis experiments showed similar number of cells/field that migration toward DMEM (control, data not shown), indicating that AMF plays a role as chemoattractant on MSCs. The most significant AMF-dependent migration of MSCs was observed at doses ranging from 0.5 µg/ml to 1 µg/ml (p<0.01) regardless of MSC sources (Figure 1B–D). Higher rAMF concentration (5 µg/ml or 10 µg/ml) were unable to induce *in vitro* chemotaxis.

**Figure 2:**
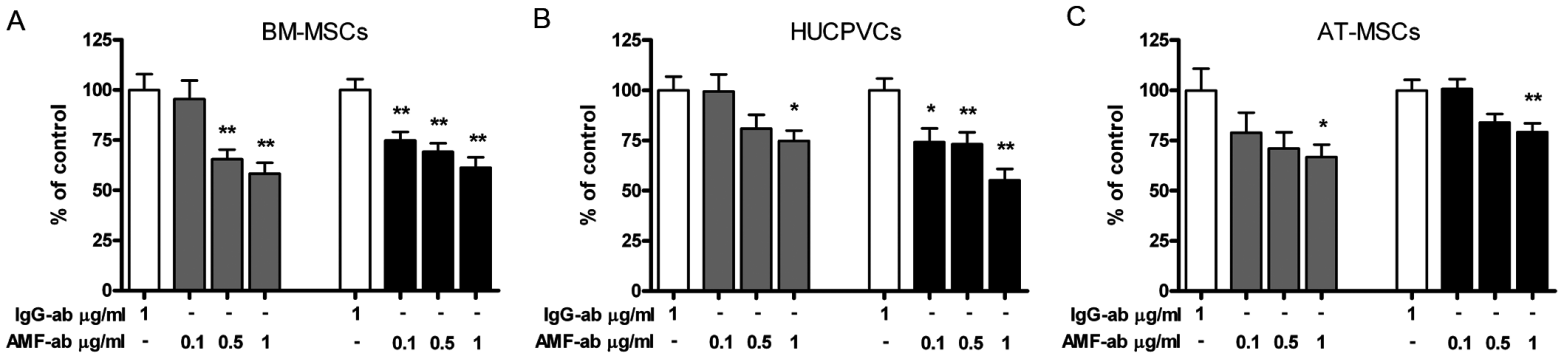
AMF produced by HCC exerts a potent role as chemotactic protein. Cell migration of BM-MSCs (A), HUCPVCs (B) or AT-MSCs (C) towards TCM derived from HuH7 (gray bars) or HC-PT-5 (black bars) pretreated with anti-AMF-Ab (AMF-ab) or isotype control (IgG-ab). Results were expressed as percentage of control (isotype control) ±SEM. *p<0.05 and **p<0.01 vs isotype control (ANOVA and Dunnett's comparison test). Results are representative of 3 independent experiments.

To examine whether AMF produced by HCC might affect MSC migration, TCM obtained from HuH7 or HC-PT-5 tumors were preincubated with a polyclonal antibody against AMF (AMF-Ab). Neutralization of AMF with the AMF-Ab reduced TCM capacity to induce MSC chemotaxis. At 1 µg/ml of the antibody we observed 20% to 40% reduction in TCM capacity to stimulate MSC migration regardless of MSC source, suggesting a significant and wide AMF effect on MSC chemotaxis (Figure 2A–C). These data demonstrated that AMF exerts a potent chemotactic effect over MSCs of different origins.

### AMF enhances matrix metalloproteinase (MMP) activity of MSCs

**Figure 3:**
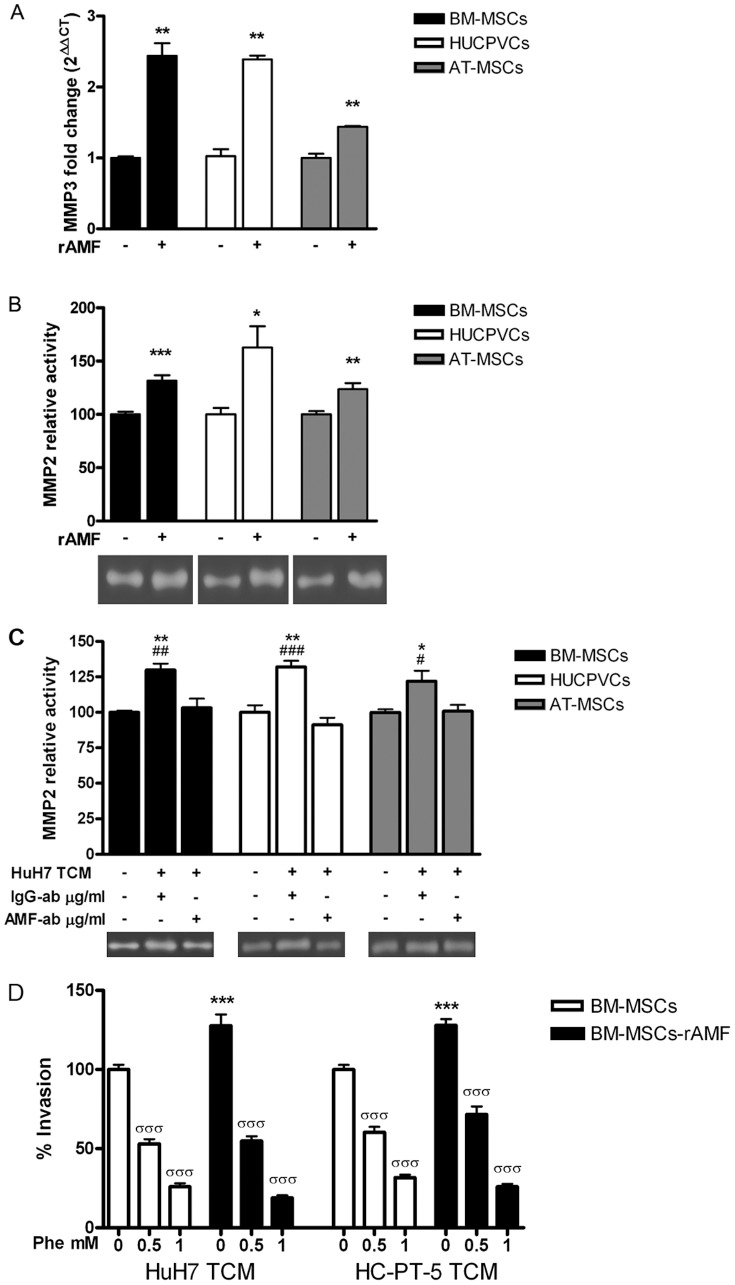
MMP3 expression and MMP2 activity is induced in MSCs by rAMF. A) Analysis of MMP3 expression by qRT-PCR in BM-MSCs (black bars), HUCPVCs (white bars) or AT-MSCs (gray bars) stimulated with 1 µg/ml of rAMF. **p<0.01 vs unstimulated cells (DMEM, unpaired Student's t test). B) MMP2 activity was evaluated by zymography in supernatants of BM-MSCs (black bars), HUCPVCs (white bars) or AT-MSCs (gray bars) pre-stimulated with 1 µg/ml of rAMF. Band intensity of 3 independent experiments was detected by densitometric evaluation and plotted as MMP2 relative activity. *p<0.05, **p<0.01 and ***p<0.001 vs untreated cells (DMEM, ANOVA and Tukey's comparison test). One representative image of the zymography is shown. C) MMP2 activity was evaluated by zymography in MSCs (BM-MSCs, black bars; HUCPVCs, white bars; and AT-MSCs, gray bars) culture supernatant stimulated with TCM from HuH7 cells. TCM from HuH7 cells was blocked with anti-AMF antibody (AMF-ab) or isotype control (IgG-ab). Band intensity of 3 independent experiments was detected by densitometric evaluation and plotted as MMP2 relative activity. One representative image of the zymography is shown. *p<0.05 and **p<0.01 vs DMEM (ANOVA); #p<0.05, ##p<0.01 and ###p<0.001 vs AMF-blocked TCM from HuH7 (HuH7 TCM+/AMF-ab+, ANOVA and Tukey's comparison test). D) Invasion capacity of BM-MSCs (white bars) or stimulated with rAMF (black bars) to type IV collagen using TCM from HuH7 or HC-PT-5 preincubated with different doses of the MMP inhibitor 1,10 phenantroline (Phe). ***p<0.001 vs without stimulation with rAMF and σσσp<0.001 vs without preincubation with Phe (ANOVA). Results are representative of 3 independent experiments.

Previous studies have shown that MSC migration depends on the activity of MMPs. Based on that, we further assessed MMP3 mRNA levels. MMP3 transcripts showed a 1.4 to 2.4-fold increase in MSCs incubated with rAMF compared to untreated (Figure 3A). We have previously reported that BM-MSC stimulated with HCC CCM showed increased MMP2 activity [19]. Therefore, we decided to analyze whether AMF might be involved in the enhancement of MSC MMP2 activity upon stimulation with HCC CM. To test this hypothesis, MMP2 activity was measured in MSCs culture supernatant with or without prestimulation with 1 µg/ml of rAMF. Gelatinolytic activity corresponding to MMP2 was detected in supernatants from BM-MSCs and also from HUCPVCs and AT-MSCs. Moreover, MMP2 activity was significantly enhanced when different MSCs were stimulated with rAMF (Figure 3B). Finally, enhancement in MMP2 activity in MSCs from different sources due to HCC stimulation was found to be abolished when HuH7 TCM was preincubated with anti-AMF antibody (Figure 3C). In addition, we performed cell invasion assay using polycarbonate membrane precoated on top of the membrane with type IV collagen. In agreement with zymography experiments, stimulation with rAMF increased the invasion capacity of MSCs across collagen (Figure 3D). As expected, the MMPs inhibitor significantly decreased the invasion capacity of MSCs. We observed that the MMPs inhibitor did not modify the migration capacity of rAMF-stimulated MSCs (not shown). From these results we conclude that AMF produced by HCC likely mediates the enhancement in MMP2 activity observed in MSCs when these cells are exposed to tumor conditioned medium.

### AMF enhances BM-MSC migration by stimulating endothelial cell adhesion and modulating relevant genes

**Figure 4:**
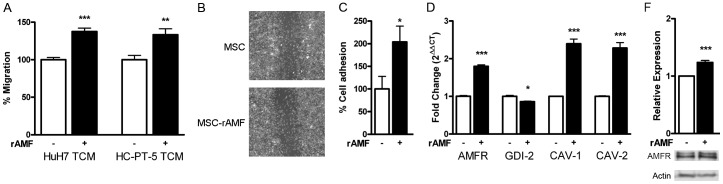
rAMF increases the in vitro chemotaxis of MSCs towards HCC and their adhesion to endothelial cells. A) Pretreatment of BM-MSCs with 1 µg/ml rAMF (black bars) increases chemotaxis towards TCM derived from HuH7 or HC-PT-5 cells compared to untreated cells (white bars). B) Wound-healing assay of MSCs after pretreatment with rAMF or control (DMEM). Representative images were taken 24 hours after scratching. C) Adhesion to HMEC-1 endothelial cells was increased in BM-MSCs exposed to rAMF. D) Expression of AMF receptor (AMFR), GDP dissociation inhibitor 2 (GDI-1), caveolin-1 (CAV-1) and caveolin-2 (CAV-2) by qRT-PCR. *p<0.05, **p<0.01 and ***p<0.001 vs untreated cells (DMEM, white bars, unpaired Student's t-test). E) AMFR expression was increased in AMF-treated MSCs evaluated by western blot.

Potent and specific MSC migration toward HCC is critical for their efficiency as cell carriers of therapeutic drugs. After observation that AMF is a chemotactic factor for MSCs, we decided to study whether the potentiation of this axis could increase the degree of MSC migration. To this end, we tested the effect of rAMF pretreatment on MSC migration towards HCC. Indeed, rAMF pretreatment induced a 40% increase in BM-MSC migration toward HCC TCM, obtained from either HuH7 or HC-PT-5 cells (Figure 4A). We also performed a wound-healing assay and observed that overnight rAMF pretreatment did not modify MSC general motility (Figure 4B) indicating that rAMF pretreatment increase specific chemotaxis towards HCC. Regarding their recruitment into tumors, adhesion of MSCs to endothelial cells is considered a crucial event for their efficient arrest at tumor vasculature, required for their subsequent transmigration. We observed that pretreatment of MSCs with rAMF resulted in a 2-fold enhancement in BM-MSC adhesion to human endothelial cells HMEC-1 (Figure 4C). In order to elucidate the molecular mechanisms behind the increased chemotactic and adhesion properties induced by rAMF-pretreatment on BM-MSCs, genes related with the AMF-AMFR pathway were studied. As shown in Figure 4D, a 1.8-fold induction of AMF receptor mRNA was observed in BM-MSCs when they were exposed to rAMF when compared to control. Additionally, mRNA levels of caveolin-1 and caveolin-2 were 2.4-fold and 2.3-fold increased respectively, while GDI-2 expression was reduced 10% after rAMF pretreatment (Figure 4D). Moreover, rAMF treatment induced the expression of AMFR (Figure 4E).

### rAMF increases the *in vivo* homing of MSCs toward HCC

**Figure 5:**
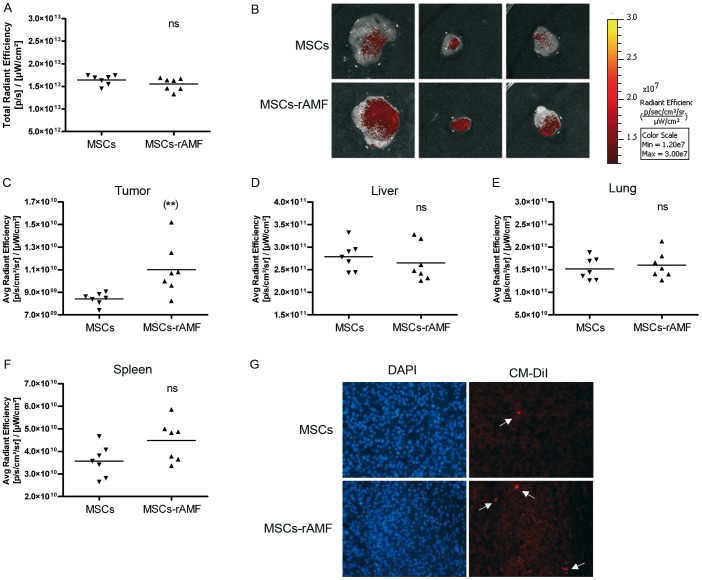
rAMF increases the in vivo migration and anchorage of MSCs to HCC tumors. BM-MSCs prestimulated with 1 µg/ml of rAMF were labeled with DiR and CMDiI cell trackers and IV injected in SC HuH7 tumor-bearing mice. After 3 days, tumors were removed and exposed to FI. A) Total FI was calculated by measuring the region of interest (ROI) for all the tissues isolated and the results were expressed as total radiant efficiency. ns, non significant. B) Representative tumor images of mice inoculated with rAMF-prestimulated BM-MSCs (MSC-rAMF) or unstimulated cells (MSCs). Images represent the average radiant efficiency. Region of interest (ROI) was calculated for the isolated tumor (C), liver (D), lung (E) and spleen (F) and the results were expressed as the average radiant efficiency. **p<0.01 vs unstimulated BM-MSCs (unpaired Student's t-test). G) Microscopic analysis of transplanted CM-DiI-labeled MSCs (red signal indicated by arrows) and DAPI staining in frozen sections of tumors. Magnification ×200.

In order to establish AMF role *in vivo* DiR and CM-DiI stained BM-MSCs were prestimulated or not with rAMF followed by their IV administration in mice carrying SC established HuH7 tumors. Three days later, mice were sacrificed and the tumor associated fluorescence was analyzed. The total FI in both groups of animals were similar, indicating no differences in the quantity of injected BM-MSCs (Figure 5A). We found that tumors from animals injected with AMF-pretreated BM-MSCs showed higher signal in comparison with control mice (Figure 5B and C). Interestingly, mice that received BM-MSC pretreated with rAMF did not show increased signal in liver, lung or spleen (Figure 5D–F), indicating a specific increased recruitment of BM-MSCs in tumor microenvironment. The presence of CMDiI-stained BM-MSCs inside the tumor mass was confirmed by cell visualization under fluorescence microscopy (Figure 5G).

**Figure 6:**
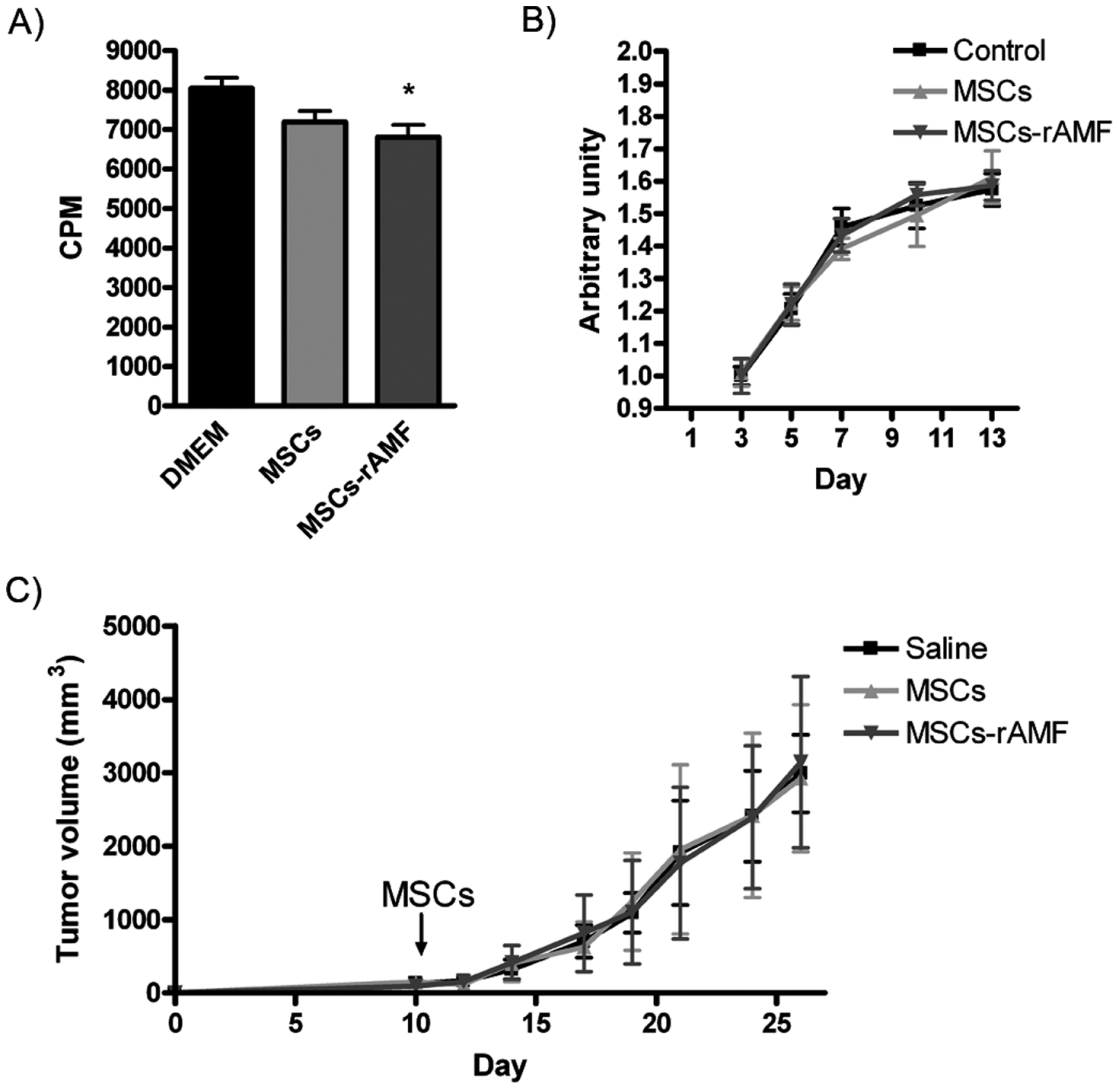
AMF treatment does not modify MSC effect on tumor growth. A) *In vitro* proliferation of HuH7 cells exposed to MSCs, AMF-pretreated MSCs (MSC-rAMF) or unexposed cells (DMEM). *p<0.05 vs DMEM (ANOVA and Tukey's comparison test). B) Multicellular spheroid growth composed by HCC tumor cells, hepatic stellate cells and endothelial cells (control) or also by MSCs or MSCs prestimulated with rAMF (ANOVA and Tukey's comparison test). C) *In vivo* tumor growth of SC HuH7 (saline) and also IV injected with MSCs or AMF-pretreated MSCs (ANOVA and Tukey's comparison test).

Previous studies have shown that MSC homing to the tumor niche may serve as a process by which the growing tumor take advantage from host cells to increase their growth [23]. *In vitro* studies indicated that HuH7 HCC cells exposed to CCM from MSC pretreated with rAMF did not enhance cell proliferation compared to unexposed cells or to HuH7 cells exposed to CCM from untreated MSCs (Figure 6A). Moreover, pretreatment of MSCs with rAMF did not affect the *in vitro* growth of multicellular spheroids composed of HuH7 HCC cells, hepatic stellate cells LX-2 and HMEC-1 endothelial cells (Figure 6B). Finally, AMF-prestimulated MSCs did not enhance tumor growth compared to control tumor-bearing mice (saline) or to the group of mice administered with unstimulated MSCs (Figure 6C). These studies indicate as a whole that AMF promotes MSC homing to the HCC niche without affecting tumor growth.

## Discussion

It is known that MSCs have the ability to migrate and engraft into tumors and it is generally believed that this property is influenced by factors produced by tumor cells and their microenvironment. Factors such as SDF-1, IL-8, IL-6 or MCP-1 which are released by HCC cells or by other tumor stroma components, have been described as chemoattractants for MSCs [6]. However, there is a lack of conclusive data confirming the role of any of these factors in the recruitment of MSCs towards HCC.

We have herein demonstrated for the first time that AMF, previously found to be produced by HCC [24], is a chemoattractant factor for MSCs. We showed that a concentration of 1 µg/ml of AMF was sufficient to induce MSCs migration, while higher concentrations of this protein were found to be necessary to induce HCC cell migration [14]. It is worth noting that AMF is not only secreted by SC tumors derived from either HCC cell lines or HCC patient samples, but their levels are greater in TCM when compared to CCM. This is consistent with our previous reports showing differences in the *in vitro* migration capability of BM-MSCs towards TCM when compared to CCM [19]. Thus, our results underline the importance of using experimental models better mimicking the tumor behavior which would be observed in patients.

Several *in vitro* studies have demonstrated that exogenous AMF stimulates migration of human cancer melanoma, fibrosarcoma and HCC cells as well as human umbilical vein endothelial cells (HUVECs) [7], [13], [25], [26]; however, this is the first report demonstrating its chemoattraction properties over MSCs. We have found that AMF is involved in the migration of MSCs of different sources, BM (BM-MSCs), perivascular cells from umbilical cord (HUCPVCs) and adipose tissue (AT-MSCs), towards HCC. However the specific blockage of the AMF present in the TCM derived from HCC is not sufficient to completely abolish MSC migration towards HCC, probably due the presence of several other cytokines/chemokines in this type of tumor.

One of the key steps in the transmigration process across the basement membrane requires the proteolytic activity of metalloproteinases. It has been previously shown for tumor cells that AMF-induced motility is mediated by upregulation of MMP2 and MMP3 [13], [14], [16]. In the present work we demonstrated that AMF has the ability to increase the expression of mRNA MMP3 in MSCs. This matrix metalloproteinase degrades fibronectin, laminin and type IV collagen, critical for cell invasion and proteolysis of the extracellular matrix. Regarding MMP2, we have previously reported that MSCs exposed to CM derived from HCC cell lines increased their activity [19]. Previous reports described the induction of MMP2 activity on MSCs after the exposure to several cytokines. Moreover, and similarly to MMP3, MMP2 activity has been shown to be necessary for cell invasion across the basement membrane and that its inhibition reduces transendothelial migration of MSCs [27]–[29]. In this report, we showed for the first time that AMF induces MMP2 activity and, consequently, their invasion capacity *in vitro*. Furthermore, MMP2 enhanced activity observed upon HCC CM stimulation depends on AMF. Thus, AMF seems to be relevant in MSC migration towards HCC since the blockage of AMF decreased MMP2 activity and migration *in vitro*. However, the use of MMPs inhibitors did not affect the migratory capacity of MSCs *in vitro*.

Although there are some promising results with MSCs genetically modified as a therapeutic option for HCC [30], [31], there is a need to enhance the efficacy of MSC migration towards HCC microenvironment. Considering the capacity of AMF to induce chemotactic effects on MSCs, we decided to test whether AMF pretreatment was capable of increasing MSC migration towards tumor microenvironment. We demonstrated that pretreatment with rAMF significantly increased MSC migration towards HCC *in vitro* and *in vivo*. It has been previously reported that MSC motility *in vitro* was induced after stimulation with different cytokines [27], growth factors [32], or chemokines such as CXCL7 [33] or SDF-1 [34]. However, this is the first report demonstrating an increased in MSC migration towards HCC *in vivo*, after a simple priming treatment of MSCs with rAMF.

In cancer cells, it has been observed that AMF-induced migration is mediated by its interaction with AMF receptor (AMFR) on cell surface [25]. AMFR has been found stably linked to caveolae in the plasma membrane caveolin-1, a caveolar coat protein that has been described as a negative regulator of caveolae-mediated endocytosis of AMFR in the endoplasmic reticulum [10]. According to this, we found that rAMF treatment induced AMFR and caveolin-1 and -2 expressions, suggesting their role in the maintenance of the receptor on the cell surface. Moreover, in cancer cells AMF enhances integrin β1 activity leading to activation of mitogen activated protein kinase (MAPK) and Rho pathways [13]. Small GTPases are largely involved in motility and cell adhesion due to their role in cytoskeleton organization. GTPase activity is regulated by GTPase-activating proteins (GAPs) and GDP dissociation inhibitors (GDIs). In bladder cancer, the expression of Rho GDP dissociation inhibitor (GDI) β (GDI2) is diminished in cells with higher motility indicating a possible role as suppressor of migration. However, other reports indicated that GDI-2 is upregulated in tumors with a more aggressive phenotype [35], [36]. We herein observed that rAMF pretreatment resulted in reduced GDI-2 mRNA levels, suggesting a role of this protein as inhibitor of migration.

It is noteworthy that the AMF-induced migration as well as AMFR's role in metastasis has been extensively described in tumor cells. There is a previous report indicating that overexpression of AMF in NIH-3T3 fibroblasts induces malignant transformation [37]. For that reason, we decided to pretreat MSCs instead of inducing AMF expression in these cells. Moreover, the effect of MSCs on tumor growth remains controversially and seems to depends on tumor models [6]. Our previous results showed that MSCs do not affect neither HuH7 cell proliferation nor HuH7 tumor growth [19]. Our recent results demonstrated that AMF priming on MSCs does not affect/influence tumor development.

Taking our results altogether, we can conclude that AMF likely plays a critical role in the HCC recruitment of MSCs. Furthermore, AMF-priming of MSCs could also be beneficial for increasing MSC migration towards HCC in therapeutic interventions.
